# Efficacy of fresh packed red blood transfusion in organophosphate poisoning

**DOI:** 10.1097/MD.0000000000006375

**Published:** 2017-03-24

**Authors:** Hang-xing Bao, Pei-jian Tong, Cai-xia Li, Jing Du, Bing-yu Chen, Zhi-hui Huang, Ying Wang

**Affiliations:** a First Clinical Medical College of Zhejiang Chinese Medical University; b Zhejiang Provincial Hospital of TCM; c Department of Laboratory Medicine, Zhejiang Provincial People's Hospital, People's Hospital of Hangzhou Medical College; d Department of Transfusion, Zhejiang Provincial People's Hospital, People's Hospital of Hangzhou Medical College, Hangzhou; e Institute of Neuroscience and Institute of Hypoxia Medicine, Wenzhou Medical University, Wenzhou; f Department of Transfusion, Lishui People's Hospital, Lishui, Zhejiang, China.

**Keywords:** atropine, cholinesterase, fresh red blood cells, organophosphate, transfusion

## Abstract

The mortality rate caused by organophosphate (OP) poisoning is still high, even the standard treatment such as atropine and oxime improves a lot. To search for alternative therapies, this study was aimed to investigate the effects of packed red blood cell (RBC) transfusion in acute OP poisoning, and compare the therapeutic effects of RBCs at different storage times.

Patients diagnosed with OP poisoning were included in this prospective study. Fresh RBCs (packed RBCs stored less than 10 days) and longer-storage RBCs (stored more than 10 days but less than 35 days) were randomly transfused or not into OP poisoning patients. Cholinesterase (ChE) levels in blood, atropine usage and durations, pralidoxime durations were measured.

We found that both fresh and longer-storage RBCs (200–400 mL) significantly increased blood ChE levels 6 hours after transfusion, shortened the duration for ChE recovery and length of hospital stay, and reduced the usage of atropine and pralidoxime. In addition, fresh RBCs demonstrated stronger therapeutic effects than longer-storage RBCs.

Packed RBCs might be an alternative approach in patients with OP poisoning, especially during early stages.

## 1 Introduction

Organophosphate (OP) compounds are diverse group of human-made chemicals with a potent toxicological threat. Several pesticides, rodenticides, fungicides are made of OPs, such as parathion, malathion, and dimethoate. OPs are frequently used in agriculture, home, gardens, veterinary practice, and intentionally used in suicides. Therefore, acute and chronic, due to its availability and toxicity, OPs are one of the most common causes of poisoning worldwide, especially in Asia.^[1]^ Actually, acute self-poisoning with OP pesticides occurs frequently in rural Asia, and causes thousands of deaths every year.^[2,3]^

OPs can be absorbed by all routes, including the respiratory tract, alimentary tract, and dermal integuments. The toxic mechanism of OPs is the interaction with and irreversibly inhibition of acetylcholinesterase (AChE), and pseudocholinesterase (PChE, or butyrylcholinesterase, BChE). This lead to accumulation of endogenous acetylcholine (ACh) concentration at synapses with resultant overstimulation of neurotransmission. Both muscarinic ACh receptors and nicotinic ACh receptors can be affected and may even lead to death due to pulmonary edema, cerebral edema, and respiratory paralysis.^[4,5]^ Standard therapies include the resuscitation, antidote administration, gastric lavage, and/or activated charcoal and supportive care.^[6]^

Despite the use of antidotes and intensive care, the high mortality rate associated with OP poisoning necessitates new alternative treatments.^[7]^ Traditional treatment approaches with oximes have limited success according to a large randomized trial^[8]^ and several systematic reviews.^[7,9,10]^ Thus, the search for effective and alternative treatments continues. Red blood cells (RBCs) transfusion as a main supply of AChE has been posed as an alternative therapy. The effect and mechanism of action, however, remains unclear. In this study, we found that RBC transfusion particularly use of fresh RBC was a suitable supplement containing active AChE. This treatment approach could promote cholinesterase (ChE) restoration, and help to improve clinical symptoms of patients with OP poisoning.

## 2 Methods

This prospective, randomized study began following the approval of the Ethics Committee of Zhejiang Provincial Hospital of TCM, and the Ethics Committee of Zhejiang Provincial People's Hospital. Patients who were diagnosed with OP intoxication in an emergency medicine clinic in the Zhejiang Provincial Hospital of TCM, and the Zhejiang Provincial People's Hospital from January 1, 2014 to January 1, 2016, were included in this study. The diagnosis of acute OP intoxication was based on^[11]^: a history of ingestion; characteristic clinical signs and symptoms such as salivation, lacrymation, convulsion, vomiting, myosis, bradycardia, bronchial secretion, and respiratory failure; marked improvement in myosis, salivation, sweating, and heart rate increase after atropine treatment; serum ChE activity less than 2000 KU/L (normal 4000–11000 KU/L). The patients who had additional carbamate poisoning with preexisting severe chronic disease and exposure time to OP intoxication longer than 3.5 hours were excluded from this study.

Standard patient data including demographic information, history, time, and amount of OPs ingested, blood ChE levels were recorded. ChE levels were determined via Olympus AU2700 Spectrophotometric Analyzer using commercial kits (Beckman Coulter, Tokyo, Japan).

All patients were treated with a classic OPs intoxication treatment including gastric lavage, intravenous (IV) atropine (starting at 1 mg/kg per day), pralidoxime (IV, 2 g single dose) and supportive care such as mechanical ventilation if necessary.

Patients were divided randomly into transfusion or nontransfusion groups. In the RBC transfusion group, packed RBCs were transfused in 3 hours after poisoning. Ten hours after toxication, if the patient is still not atropinization or had a low blood ChE level, another 200 to 400 mL of packed RBCs were transfused. All packed RBCs were administrated within 72 hours after intoxication. Packed RBCs were divided acording to the storage days. Fresh RBCs refers to storage less than 10 days (including 10 days). Longer-storage RBCs refers to storage for more than 10 days, but less than 35 days (in China, the validate length of RBCs storage is 35 days with CPDA-1, according to the “Law of the People's Republic of China on blood donation”).

A statistical analysis was performed using ANOVA with pair-wise comparisons or Chi-square test. Statistical significance was defined as *P* < 0.05 for all tests.

## 3 Results

### 3.1 Fresh RBC transfusion significantly improved ChE recovery in OP poisoning patients

A total of 80 patients was included in the study. Fresh RBC transfusions were applied to 27 patients. Longer-storage RBC transfusions were applied to 23 patients. Thirty patients did not recieve transfusions. The mean age, sex, mean plasma AChE levels in realtion to the severity of poisoning, and time to emergency are shown in Table 1. No significant difference was found between groups. All of the patients had OP compound exposure history due to an accident or a suicide attempt. All exposure routes were oral. Twenty-two patients were poisoned with dichlorvos, 17 with malathion, 13 with dimethoate, 12 with methamidophos, 9 with parathion, and 7 with trichlorfon. The smallest intoxication dose was 10 mL trichlorfon, the largest dose was 500 mL dichlorvos. The duration from intoxication to the emergency was average 2 hours, the shortest was 0.5 hours, the longest was 3.4 hours.

**Table 1:**
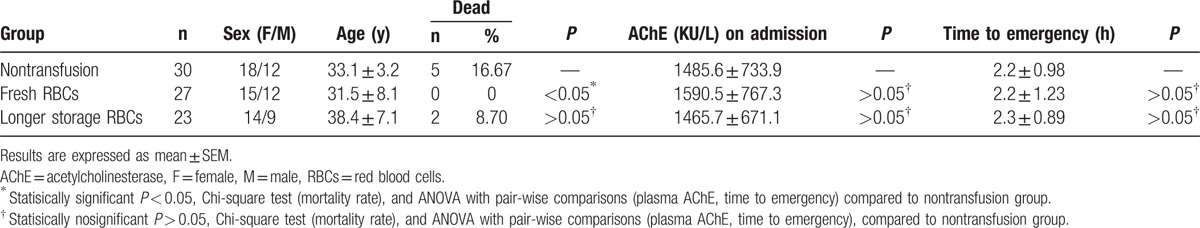
Comparison of the groups for demographic characteristics, mortality rate, and plasma AChE.

**Figure 1:**
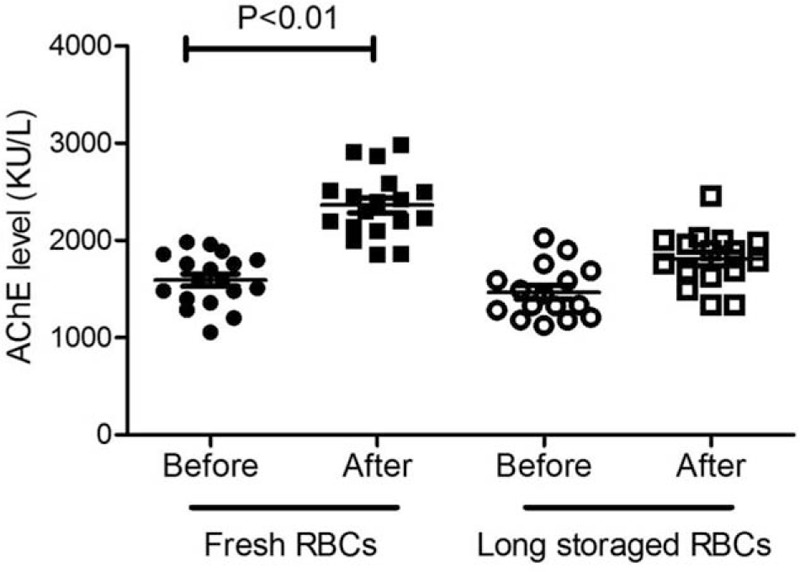
Fresh RBC transfusion significantly improved ChE levels of the patients. Blood ChE levels before and after RBC transfusion (fresh or long-storage RBCs respectively) 6 hours were shown. *P* < 0.01, ANOVA with pair-wise comparisons, compared between before and after RBC transfusion groups.

In order to follow AChE elevation effects by RBC transfusion, blood levels of AChE were measured level before and 6 hours after RBC transfution. In the fresh RBC transfusion group, blood levels of AChE after transfusion was significantly increased (Fig. 1). This significant increase was due to AChE supplementation from the transfused fresh RBCs. In the longer-storage RBC transfusion group, however, blood levels of AChE increased, but without statistical significance (Fig. 1). This may have been due to the reduction of AChE during storage time. These results suggest that fresh RBC transfusions significantly improved AChE levels in patients with OP poisioning.

### 3.2 RBC transfusion shortened the time for ChE recovery to the normal level in OP poisioning patients

Blood ChE elevation is an important indication of recovery in patients with OP poisoning. In this study, the average time for ChE recovery to 70% (2800 KU/L) and 90% (3600 KU/L) of normal levels were recorded and compared. As shown in Table 2, both fresh and longer-storage RBC transfusions significantly shortened the time for ChE recovery to normal levels. In particular, fresh RBC transfusions had a better effect than longer-storage RBC transfusions. These results suggest that RBC transfusion significantly shortened the time for ChE recovery to a normal level in OP patients.

**Table 2:**
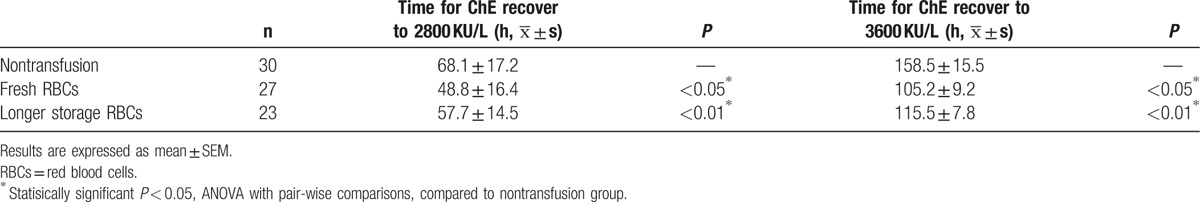
Packed RBC transfusion shortened duration time for ChE recovery in OP poisoning patients.

### 3.3 RBC transfusion reduced the usage of atropine and shortened the duration of atropine in OP poisioning patients

Atropine usage and durations reflect the severiety and progression of the intoxication. As shown in Table 3, fresh RBC transfusion significantly reduced both the usage of atropine per day and total, compared with the no transfusion group. Longer-storage RBC transfusion reduced the total amount of atropine used (Table 3). Moreover, both fresh and longer-storage RBC transfusions significantly shortened the duration of atropine use (Table 3). In addition, fresh- and longer-storage RBC transfusions significantly shortened the duration of pralidoxime use (Table 4). Fresh-storage RBCs also significantly reduced the number of days in hospital (Table 4). The Acute Physiology and Chronic Health Examination (APACHE) II score is a well-validated scoring system^[12]^ used to predict severity in patients requiring intubation. No significance difference, however, was observed in the APACHE II scores between groups (Table 4). Taken together, these results suggest that RBC transfusion reduced useage and duration of in OP poisioning patients.

**Table 3:**
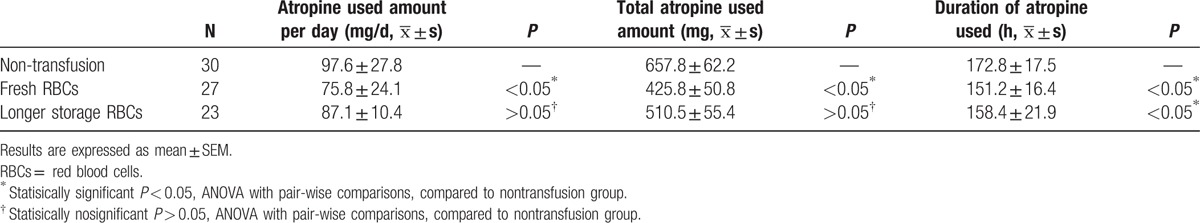
Packed RBC transfusion reduced atropine usage in OP poisoning patients.

**Table 4:**
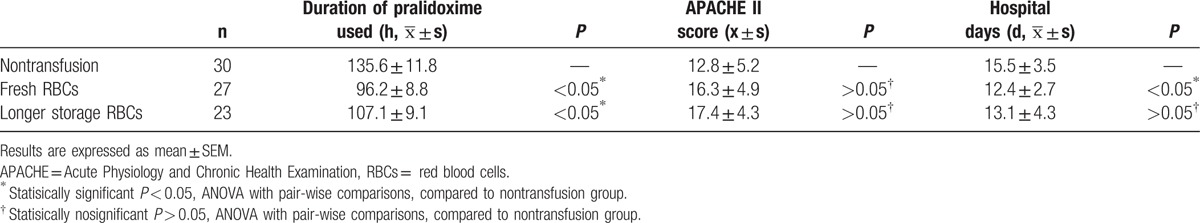
Effect of packed RBC transfusion on pralidoxime usage, intubation scores, and hospital days in OP poisoning patients.

## 4 Discussion

In this study, we found that both fresh and longer-storage RBCs: significantly increased blood ChE level 6 hours after transfusion; shortened the duration of ChE recovery and hospital days; and reduced the atropine and pralidoxime usage. Thus, RBC transfusion, especially, use of fresh RBCs as a suitable source of active AChE, could promote ChE restoration, and help to improve clinical symptoms.

OP poisoning may be result from both accidental and purposeful intake. In acute or chronic, OP poisoning is potentially fatal, and with a risk of remote consequences.^[13]^ As a strong inhibitors of ChE, OPs cause acetylcholine accumulates, acetylcholine receptors paralysis, leading to muscarnic, nicotinic, and central nervous system symptoms.^[14]^ In severe poisoning, it needs 4 weeks for ChE recovery. If low ChE level lasts, it might cause intermediate syndrome, leading to respiratory muscle paralysis. In this study, we found 200 to 400 mL RBC transfusion could significantly increase ChE activity. Longer-storage RBCs transfusion demonstrated the similar but smaller improvement effect. Thus RBC transfusion may improve the clinical therapeutic effects on OPs poisoning patients. These results present an additional therapeutic option in patients with reduced ChE, especially when atropine or specific antidote treatment are ineffective.

The 2 types of ChEs are AChE and BChE, both concentrated in the circulation. Through attachment to free OPs in the blood, they will be metabilised, to prevent severe damage to the central nervous system. AChE is manily concentrated on RBCs menbranes and BChE is primarily enriched in blood plasma. AChE is essential for enzyme activity since it has a higher specificity to ACh than BChE. Increasing AChE activity over 30% leads to normal neuromuscular transmission, and can improve the overall outcome.^[15]^ It is possible that the allogenic RBCs, a source rich in AChE, deliver extra erythrocyte ChE, and become potential target substrates for OPs. Therefore RBC transfusion can not only substitute circulating ChE, but also prevent OPs from entering the central nervous system and muscle tissue.

It has been reported that during RBCs storage, AchE activity remains constant until day 7, and reduced dramatically at day 45.^[16,17]^ Here we found fresh packed RBCs did have a better effect than longer-storage RBC. The fresh RBCs which storaged less than 10 days significantly improved blood ChE activity and clinical symptomes in OP poisoning patients. Thus, in an emergency, both fresh and longer-storage RBCs could be used to supply AChE, leading to improvement. Due to the decline during storage, however, the fresh RBCs would be the better choice.

Previous studies have reported that whole blood transfusion^[13]^ or fresh frozen plasma transfusion^[18]^ could restore enzymatic function by its scavenging effect. Packed RBC transfusion has advantages over those transfusions, since the small total volume administered to patients could avoid risks of overloading, fever, or allergy by components in the serum. Specific administration of human BChE substitutes^[19]^ could also restore enzymatic function.^[20]^ However, due to its high costs and the large quantity of products needed, it is not currently feasible. Though binds OP compounds stoichiometrcally, RBC-AChE function as a natural bioscavenger, to effectively inactivating OPs, then make RBC transfusion the possibility as an optimal alternative approach.

A limitation of this study includes that blood ChE levels do not fully represent the ChE levels in the sympathetic ganglia or gray matter in the central nervous system. Although ChE levels in the blood and nervous system could supplement one another, the substantial increase of ChE levels in the presynaptic membrane warrants further study.

In summary, early blood transfusion in patients with OP poisoning can effectively reduce the extent and progression of toxic symptoms, especially when oximes are unavalilable.
